# 

**Published:** 1868-09

**Authors:** 


### CONTENTS.

ORIGINAL COMMUNICATIONS.

Professional Bickerings, ... 355
Alveolar Abscess ... 358
Microscopy of the Teeth ... 363
Resistance of the Dental Pulp to the Action of Arsenious Acid ... 370
Advantages and Disadvantages of Aluminum as Applied to Dentistry ... 371

SELECTIONS.

The Probable Causes of Malaria and Epidemical Diseases ... 373
A New Process of Mummification ... 384
Correspondence ... 389
Notice ... 390

EDITORIAL.

Richardson on Nitrous Oxyd ... 391
American Dental Association ... 394
Appreciated ... 396
Ohio Dental College Session ... 396
Trichina Spiralis ... 397
Bibliographical ... 397

